# Sharing on platforms: Reducing perceived risk for peer‐to‐peer platform consumers through trust‐building and regulation

**DOI:** 10.1002/cb.2075

**Published:** 2022-06-18

**Authors:** Sarah Marth, Barbara Hartl, Elfriede Penz

**Affiliations:** ^1^ Institute of Marketing and Sales University of Applied Sciences Wiener Neustadt Wiener Neustadt Austria; ^2^ Institute for Marketing and Consumer Research Vienna University of Economics and Business Vienna Austria; ^3^ Institute for International Marketing Management Vienna University of Economics and Business Vienna Austria

## Abstract

Sharing a flat with strangers is no longer hypothetical but well accepted by many consumers who participate in peer‐to‐peer (P2P) services. Online P2P sharing platforms act as intermediaries between providers and consumers who do not know each other personally. Sharing via platforms entails a certain amount of risk for consumers. Thus, in order to attract consumers, platforms need to apply mechanisms to reduce the perceived risk of potential consumers. In a prestudy and two experimental online surveys, the current research investigates whether trust‐building measures and regulation mechanisms presented on a website can reduce the potential consumers' level of perceived risk and increase their willingness to participate in a platform's sharing offer. First, an analysis of existing P2P accommodation platforms showed a lack of regulation mechanisms. Second, the manipulation of information on P2P accommodation platforms' websites in two online experiments revealed that regulation mechanisms led to lower perceived risk and a higher intention to engage in sharing. However, commonly used trust‐building measures on P2P accommodation platforms show no significant effect on risk perception and the intention to engage in sharing in the present study. We point out relevant managerial possibilities to minimise the perceived risk of potential consumers of P2P platforms.

## 1 INTRODUCTION

In the last decade a new method of peer‐to‐peer (P2P) exchange has developed, triggered by economic crises, increased environmental awareness and facilitated by a number of technological innovations, which have simplified sharing of goods and services (cf. Hamari et al., 2015; Oskam & Boswijk, 2016; Zervas et al., 2017). In the academic literature, this exchange is referred to as a ‘sharing economy’, ‘collaborative consumption’, ‘collaborative economy’ or ‘platform economy’ (Belk, 2014b; Botsman & Rogers, 2010; Kenney & Zysman, 2016; Sundararajan, 2014), which has become a phenomenon of relevance, especially in marketing, providing challenges and opportunities for business worldwide (Lim, 2020; Lim et al., 2021).In the sharing economy, people share products or properties, for instance their flat, with peers (Möhlmann, 2015). Online P2P marketplaces, so called digital ‘sharing‐economy platforms’ (cf. Dillahunt & Malone, 2015), help to coordinate sharing among strangers by facilitating connections between them. One of the most prominent examples is Airbnb, an online community marketplace facilitating short‐term rentals (Dolnicar, 2021; Karlsson et al., 2017; Lim et al., 2021; Park & Tussyadiah, 2020). In traditional marketplaces, the company typically owns the product, good or property being exchanged. In contrast, in online P2P sharing via platforms, the company involved (the platform) acts as an intermediary. It has a coordinating role instead of acting as a seller or buyer, because private persons are in possession of the good in demand. As the platform is not the owner of the good (e.g., the accommodation), but rather a mediator between two private persons, the sharing economy comes with specific risks, such as price gouging, asymmetric information and exploitation (Koopman et al., 2015; Park & Tussyadiah, 2020). Additionally, while increased internet access has enabled the sharing economy to become popular (cf. Belk, 2014a; Belk, 2014b; Schor & Fritzmaurice, 2015), the internet‐based marketplace of online P2P sharing platforms comes with additional problems, like anonymity, impersonal communication and a lack of identity verification, increasing the risk for consumers (Beldad et al., 2010; Pavlou, 2003). Consumers of a P2P platform mention concerns regarding risks, such as fraud, spam, the possible misuse of personal data as well as transaction and insurance insecurity (Constantinides, 2004; Ravenelle, 2017), as well as free‐riding (i.e., users abusing the system, cf. Hartl et al., 2016). For example, Hall (2022) reports on the lack of security and identity checks of user accounts on Airbnb. In addition, during the Covid‐19 pandemia, cases of Airbnb denying refunds for cancelled trips due to the pandemia were reported (Tims, 2021). Thus, potential consumers may be deterred by a high level of perceived risk. For P2P platforms acting in the sharing economy, dealing with risks and engaging in risk mitigation becomes highly relevant to attract new customers. Previous research (e.g., Hofmann et al., 2017) suggests that P2P platforms can decrease consumers' risk perception in two ways: the platform can either (i) take measures to increase consumers' trust in the platform or (ii) employ self‐regulated measures, such as sanctioning misbehaviour.

If P2P platforms try to mitigate the risk and want to inform potential customers about their trust and regulatory measures to decrease consumers' risk, the website of the P2P platform might be the first contact point with consumers. It informs consumers of the services and the sharing process, even before consumers interact and decide to participate in P2P sharing. To minimise the level of perceived risks in consumers who show interest, P2P platforms can either concentrate on gaining (potential) consumers' trust by applying trust mechanisms on their website or count on self‐regulation, such as control and sanctions, also displayed on the website (e.g., Grabner‐Kräuter & Kaluscha, 2003; Hamari et al., 2015; Slovic, 1987). Concerning trust, the study of Bart et al. (2005) identified different drivers of online trust, which can be implemented on a website to further facilitate trust between potential consumers and the company, such as statements concerning privacy, security, community features or an error‐free website. Another trust‐building mechanism that is often implemented in online P2P sharing platforms and announced on the website of P2P platforms are reputation systems, which can be applied in order to overcome the problem of asymmetric information and economic risks in trading with strangers via digital platforms (Resnick & Zeckhauser, 2002) and decrease the level of perceived risk in potential consumers. Reputation systems can thus act as a tool to enhance trust (Resnick et al., 2000). Additionally, platforms can add information on their websites on self‐regulated measures, such as setting up rules, controls and sanction systems for their consumers (Hofmann et al., 2017).

The current research tests the assumption that trust‐building mechanisms as well as self‐regulation impact potential consumers' risk perception and, furthermore, their willingness to participate in sharing via P2P platforms. Consumers who perceive high risks may not engage in the sharing economy, as risk perception and trust are essential aspects in purchase and transaction decisions (Ahrholdt, 2011; Grabner‐Kräuter & Kaluscha, 2003). Despite the rather large amount of academic research on regulation and trust in P2P sharing platforms, the current research links these aspects via risk perception to potential consumers' consideration of engaging in P2P sharing activities. Thus, the aim of the current research is to first investigate whether online P2P platforms apply regulation and trust‐building measures, as they are often accused of lacking regulations (cf. Cusumano, 2015; Rauch & Schleicher, 2015). Second, we examine how regulation and trust‐building measures influence potential consumers' risk perception as well as their intention to engage in online P2P sharing. Thereby, the current research fills an important gap in the research on the relationship between regulation, trust, risk perception and behavioural intention. Moreover, we provide managerial implications of possible measures online P2P platforms can take to (a) reduce potential consumers' level of perceived risk and (b) encourage them to use the platform's services. The current research concentrates on platforms in the field of accommodation sharing as an example of online P2P platforms within the sharing economy. In addition, we focus on potential consumers who consider participating in online P2P accommodation sharing as guests (as opposed to hosts) and therefore formulate the following research question: How does information about regulation mechanisms and implemented trust‐building measures on the website of a P2P accommodation platform influence the level of perceived risk of potential consumers and their intention to join the platform and use its services?

In the next section, the conceptual background of the sharing economy, risk perception, trust and regulation will be discussed. After presenting the research hypotheses, the qualitative prestudy and the main study using an experimental online survey will be elucidated, followed by the presentation of results. Finally, the results, limitations and implications of the current research will be discussed.

## 2 CONCEPTUAL BACKGROUND

The term ‘sharing economy’ is used for a multitude of activities with different social practices (Belk, 2014a), covering a range of transactions in almost all business areas, including food production (e.g., communal gardens), mobility (e.g., car sharing) and accommodation. The origins of the sharing economy can be traced back to the late 1990s when the first online platforms appeared and connected strangers worldwide (Botsman & Rogers, 2010). For example, eBay was a platform enabling private persons to sell and buy goods (Stern & Stafford, 2006). B2C renting and sharing platforms, e.g., the car‐rental platform Zipcar, as well as P2P sharing platforms, like Airbnb and Uber, followed (cf. Martin, 2016). P2P sharing platforms share the feature of enabling consumers to collaboratively make use of products for a certain period. These platforms act as an intermediary and connect individuals who have underutilised resources with people who would like to use those for a short period (Cusumano, 2015; Guttentag, 2015). Thereby, the owner of the shared good is not the platform, but a private person. Examples for such underutilised resources are cars, tools or clothes, which are only used occasionally by the owner.

Companies within the sharing economy have rapidly expanded worldwide and increased their market share “undertaking aggressive competitive moves […] both in the home country and abroad” (Parente et al., 2018, p. 53). Sharing economy business models comprise new challenges for the market place. In contrast to conventional businesses, platforms organising sharing economy activities are blamed for not offering a standardised level of service and price (Cusumano, 2015) and for lacking safeguards for consumers (Rauch & Schleicher, 2015). After an initial phase of euphoria, the sharing economy has been publicly accused of having high risks and lacking regulation (Braw, 2014; The Editorial Board, 2014), and there have been discussions about possible measures for risk mitigation. This led to the question of whether regulations protect consumers or discourage companies from actually entering the sharing economy (Koopman et al., 2015; Rauch & Schleicher, 2015) and furthermore in academic research, provoked the topic of risks for consumers (e.g., Mittendorf, 2017; Yi et al., 2020).

In the current research, we focus on perceived risk of potential consumers of online P2P accommodation sharing organised by an online platform and the main research interest of the current research is to answer how perceived risk can be mitigated by regulatory and trust‐building measures on the platforms' websites. In the case of accommodation sharing, the underutilised resources being shared are rooms, apartments, houses or any other form of living space which is not used by the owner all the time and can therefore be rented out to other people (Dolnicar, 2021; Karlsson et al., 2017; Lim et al., 2021; Park & Tussyadiah, 2020). The sharing economy enables P2P accommodation platforms to serve two consumer segments: owners of accommodation (host) offering accommodation (e.g., room, apartment) online via the platform and other people (guest) looking at the offers, booking accommodation, and paying via the platform. If P2P platforms want to engage in risk mitigation, they have to consider that both types of consumers require different regulatory and trust‐building measures as they are exposed to different risks. Therefore, the current research focuses on potential guests of P2P accommodation platforms.

### 2.1 Risk and risk perception

The perception of potential risks gains importance at an early stage in the process of accommodation sharing, when consumers interested in the service search for P2P accommodation offers. Asymmetry in the information between the potential consumers and the platform can be an initial obstacle when browsing the web for P2P accommodation platforms. The information asymmetry occurs because the platform, unlike the potential consumer, has a complete overview of all offers and full information about the processes and sharing activities happening via the platform. Additionally, the platform as the party setting up rules, for instance, in the form of general terms and conditions, has an information advantage compared with a potential consumer, who is required to invest time in order to achieve the same information level as the platform (cf. Dermawan et al., 2020). While this asymmetry is also present in offline stores, it is much more striking in a digital market environment. Yi et al. (2020) used the example of Airbnb to demonstrate that financial and privacy risks in particular decrease the chance of using the sharing offer.

By its nature, P2P accommodation platforms' service quality can only be judged after a completed transaction. Therefore, potential consumers have to depend on other indications in order to evaluate the possible risks when participating in online P2P platforms (cf. Ahrholdt, 2011). These indications can typically be found on the platforms' website, which is the main communication tool of P2P platforms. Recent research has already shown that the aesthetics of hospitality websites in the sharing economy influences customers' intention to actually book accommodation (Xu & Schrier, 2019). However, besides aesthetics and design aspects, the question remains as to which content should be implemented on a website of P2P accommodation platforms to reduce potential consumers' level of perceived risk and thus attract persons to engage in P2P accommodation sharing and if those actions differ from the actual methods currently used by online vendors.

### 2.2 Trust and regulation

One important factor relevant for reducing the level of perceived risk is increasing trust in the platform (ter Huurne et al., 2017; Viklund, 2003). In conditions of risk and uncertainty, the function of trust becomes especially valuable to economic activity (Korczynski, 2000). In the current research, we follow the definition of trust by Korczynski (2000) and Mayer et al. (1995), who define trust as the confidence that the other party will not exploit one's vulnerability.

From a marketing perspective, trust leads to a higher purchase intention and increases the likelihood of purchases (Grabner‐Kräuter & Kaluscha, 2003; Kim et al., 2008). Research on offline sales encounters revealed a high influence of people's first impression of a salesperson on their trust in that salesperson, as well as the perceived trustworthiness of the firm (Wood et al., 2008). Trust in a salesperson leads to customer loyalty (Chen & Quester, 2015). However, trust is particularly difficult to enforce if there is little personal interaction, as is the case in online P2P sharing. Platforms in the sharing economy that only operate online cannot benefit from a good first impression of the salesperson; consequently, they need to use other managerial strategies to gain consumers' trust. Sharing economy platforms may benefit from the experience of online vendors and apply trust‐building measures to their websites (Resnick et al., 2000; Resnick & Zeckhauser, 2002). Bart et al. (2005) developed a list of trust‐building measures that can be implemented on a website of a company to signal trustworthiness to potential consumers. Such trust‐building measures are, for instance, the presence of information about payment security, a consistent layout throughout the website and the presence of a support hotline. However, as described above, P2P sharing platforms differ from online vendors and the known trust‐building measures on websites may not be sufficient for P2P platforms. In addition to trust‐building measures derived from practices of online vendors, P2P sharing platforms have established reputation systems as a form of trust‐building measure in order to address the problem of risk in P2P sharing. Reputation systems collect, distribute, and aggregate peer feedback about the past behaviour of individual consumers establishing the shadow of the future by creating an expectation that other people will look back on (Resnick et al., 2000). Reputation systems, such as star ratings and comments for the host and other possible guests to read “help people decide whom to trust, encourage trustworthy behaviour, and deter participation by those who are unskilled or dishonest” (Resnick et al., 2000, p. 46) and are therefore an important source of information for potential consumers.

Another way to reduce consumers' risk perception is to establish strict regulations, for example, rules, controls and sanctions for not following the rules (Slovic, 1987). Businesses in the sharing economy are sometimes blamed for a lack of external regulations (e.g., state regulations), which results in various problems and exposes consumers to diverse risks, such as price gouging, asymmetric information and exploitation (Koopman et al., 2015). Unlike traditional businesses in the hotel sector, sharing economy businesses are usually unregistered and as such are not liable to external regulations (Yang et al., 2017). However, P2P platforms can engage in self‐regulation. For online P2P accommodation platforms, such regulations are typically presented on the website as terms and conditions and include all sorts of self‐regulation, meaning rules, controls and sanctions, which the platform as a company has determined. These regulations range from rules concerning furnishing particulars when creating an account to sanctions for misbehaviour after a shared accommodation was used.Based on this research, we assume that P2P accommodation platforms can reduce consumers' risk perception by implementing regulation as well as trust‐building measures on their website. The aim of the current research is to find out to what extent information on regulatory and trust‐building measures are present on websites of online P2P platforms and if those measures can help to reduce levels of risk perception and therefore increase the intention to engage in P2P sharing activities via an online platform. In a prestudy, we analyse the current usage of trust‐building measures and regulatory information on the website of online P2P platforms. Afterwards, in an experimental online survey, we analyse the influence of regulation mechanisms and trust‐building measures on a website on risk perception and the intention to engage in P2P accommodation sharing via a platform. Thereby, we hypothesise the following (also see Appendix 1):

Information about regulation mechanisms on the website of P2P accommodation platforms result in lower levels of risk perception, which leads to a higher intention of potential consumers to engage.

Trust‐building measures on the website of P2P accommodation platforms result in lower levels of risk perception, which leads to a higher intention of potential consumers to engage.

## 3 METHODOLOGY

The prestudy is based on a descriptive design to record trust‐building measures and regulation mechanisms in terms of rules, control and sanctions that are indicated on P2P platforms' websites. This attempt allows identification of the current measures for reducing potential consumers' perceived levels of risk regarding P2P accommodation platforms and acts as a basis for the main study. In an experimental study, we investigated whether the presentation of this information on a website influences risk perception and the intention to use the service, testing H1 and H2.

## 4 PRESTUDY: ANALYSIS OF EXISTING P2P ACCOMMODATION PLATFORMS

### 4.1 Method

#### 4.1.1 Sample

The content of four websites of P2P accommodation sharing platforms were analysed according to regulation and trust‐building measures, namely the Austrian websites of Airbnb,^1^ Wimdu,^2^ and the German websites of 9flats^3^ and gloveler.^4^ All four platforms share the same business model; that is, platforms providing a marketplace for consumers to offer accommodation and search for and book accommodation.

#### 4.1.2 Procedure

The analysis was conducted in two parts. On the one hand, the platforms' websites were searched for trust‐building measures. On the other hand, we inductively coded and analysed the terms and conditions of each platform to derive applied regulation mechanisms. Based on research by Bart et al. (2005), we created a checklist including trust‐building measures that can be implemented on a website. The checklist consisted of seven categories (privacy, security, navigation and presentation, advice, order fulfilment, community features and absence of errors), each comprised of five to twelve measures (64 measures in total, see Table 1). Two researchers coded the presence or absence of trust‐building measures on the four websites according to the checklist. Additionally, the presence of a reputation system was examined. The coding was compared and dissimilar coding was discussed until agreement was reached.

**TABLE 1:** Categories of the checklist with number of included measures, measure examples and number of occurrences on P2P accommodation platforms.

Category (Bart et al., 2005)	No of measures	Example of measure	Airbnb^a^	Gloveler^a^	9flats^a^	Wimdu^a^
Privacy	8	Information regarding security of payments is clearly presented	5	5	7	6
Security	5	There are trust seals present.	0	0	0	1
Navigation and presentation	12	The site layout is consistent across all pages	10	6	8	8
Advice	12	A toll‐free number is present for live help	7	7	5	5
Order fulfilment	9	It is possible to contact a platform's assistant through e‐mail	7	6	6	8
Community features	11	Testimonial by past consumers is present	3	3	2	5
Absence of errors	7	There were no errors or crashing	4	2	2	4
Total: 7 categories	64		36	29	30	37

^a^ Number of occurrences.

Concerning regulations ofAbrahaon the platforms, the text of the general terms and conditions was analysed following a template analysis (Brooks et al., 2015). The aim of the analysis was to identify and categorise statements about rules, controls and sanctions for guests and general consumers. Rules, monitoring and sanctions for hosts of accommodation were not part of the analysis as the current research focuses on consumers in their role as guests. Two researchers read the general terms and conditions of each website independent of each other and highlighted text passages mentioning aspects of self‐regulation (e.g., rules and sanctions). These parts were then organised into clusters and their relationship to each other was defined, resulting in a final coding scheme. In addition, the text passages, clusters and final coding schemes were compared and discussed until both researchers reached an agreement. The prestudy was conducted with the method of inductive coding, so no initial concepts were considered, but only developed in the process of reading and coding.

### 4.2 Results

The analysis of the websites concerning trust and regulation mechanisms revealed that, in general, the different categories of trust‐building measures applied by traditional online business‐to‐customer marketplaces (Bart et al., 2005) were present on websites of P2P accommodation platforms as well. However, their presence varied between the websites of the four platforms (see Table 1). Particularly striking was the small number of community features (e.g., chatroom, interaction with other consumers) used by the examined platforms. Furthermore, only a small number of external quality seals, which honour the platforms' trustworthiness, security or technical flawlessness, were found on the websites. As expected, in addition to the trust‐building measures, all websites offered a reputation system to enable consumers to rate accommodation and/or write comments about their stay.

Concerning the information on regulation displayed on the four websites, the template analysis revealed a variety of rules on all platforms, either in terms of obligations for consumers or as general rules in the form of directives and prohibitions. Given the wide range of different rules, the explicitly stated refusal of control was particularly notable. For example, the terms and conditions of 9flats stated the following: “9flats can only carry out a limited check of the data stored during the registration because the identification of persons on the Internet is limited.” Furthermore, they stated that “no control of customer ratings is performed by 9flats”, as well as no control of content uploaded by consumers (9flats, 2022). Although all platforms' terms and conditions included rules and obligations for consumers regarding different kinds of topics, the platforms clearly stated that the platform neither checks the identity of its consumers nor monitors reviews or controls other consumers' information. Only the platforms gloveler and 9flats mentioned checking the messages between consumers randomly or in the case of suspected violation of general terms and conditions. These checks should help prevent guests and hosts circumventing the platform by arranging lodging options outside the platform's system. This would lead to a loss of service fees for the platform. Although the information on the website indicates that almost no regular checks were performed by the platforms, all platforms investigated state their right to impose different sanctions. The terms and conditions mentioned the modification demands of reviews, texts and other contents, the deletion of identical content, warnings, complaints, monetary sanctions as well as the limitation of usage and (temporary) closure of a consumer's profile. The table in Appendix 2 gives an overview of the content of general terms and conditions based on the template analysis.

## 5 EXPERIMENTAL STUDY 1

Building on the results of the prestudy, the goal of the experimental study is to compare the impact of trust‐building measures and information about regulation on risk perception and potential consumers' willingness to engage in P2P accommodation platforms' sharing activity. In addition to one trust‐building measure originating from Bart et al. (2005), the impact of a reputation system is also taken into account as it could be found on each platform analysed in the prestudy.

### 5.1 Method

#### 5.1.1 Sample

A convenience sample from European countries, predominantly from Austria (78.8%), was drawn for an online survey. Out of 370 participants who finished the questionnaire, for further analysis, only participants were chosen who passed both the manipulation check on regulation as well as on trust‐building measures. Thus, the final sample consists of 151 participants (35.8% men, *M*_age_ = 24.46 years, SD_age_ = 5.45, Range_age_ = 18–51). The majority of participants either had a secondary school completion certification (49.7%) or a university degree (47.0%). 62.3% mentioned having experience with P2P accommodation platforms, 58.3% of them reported having booked accommodation at least once, and 9.3% stated having offered accommodation themselves at least once.

#### 5.1.2 Procedure

In order to identify which trust‐building measure according to Bart et al. (2005) is most important to potential consumers, a pre‐test (*N* = 66; 22.7% men, *M*_age_ = 27.68 years, SD_age_ = 6.90, Range_age_ = 20–48) was undertaken. After a short description of P2P accommodation platforms at the beginning of the questionnaire, a total list of 15 measures was presented to all participants. These 15 measures were chosen because the platforms' websites in the study varied strongly regarding presence and absence of these measures.

The participants had to choose the five most important measures to rate a platform as trustworthy and free of risks. In the next step, each participant had to rank the chosen five measures according to their importance from the most important at the top to the least important at the bottom. In addition, participants rated the importance of each of the 15 measures on a 7‐point Likert scale ranging from 1 (“absolutely unimportant”) to 7 (“absolutely important”). From the list of 15 trust‐building measures, 72.7% of participants chose Information regarding security of payments as one of the five most important measures on a website with *M*_Ranking_ = 2.42 (SD_Ranking_ = 1.24), followed by Endorsement of past users (65.2%, *M*_Ranking_ = 2.35, SD_Ranking_ = 1.34) and Informative pictures of accommodations (59.1%, *M*_Ranking_ = 3.03, SD_Ranking_ = 1.31). When it came to ranking the chosen five measures by importance, Endorsement of past users was ranked highest, followed by Information regarding security, trust seals of external validators (48.5%, *M*_Ranking_ = 2.44) and endorsement of newspapers (24.2%, *M*_Ranking_ = 2.94). Concerning participants' rating of the importance of every measure on a 7‐point Likert scale, again, information regarding security of payments was chosen as the most important (*M*_Likert_ = 6.35, SD_Likert_ = .89), followed by Informative pictures of accommodations (*M*_Likert_ = 6.11, SD_Likert_ = .95) and Endorsement of past users (*M*_Likert_ = 6.09, SD_Likert_ = .94). For a complete overview of the trust measure and participants' importance ratings, see Table 2.

**TABLE 2:** Importance of trust‐building measures.

Trust measure	Chosen as one of 5 most important by %	Rank (mean)	Importance (Likert scale) mean (SD)
Information regarding security of payments	72.7	2 (2.42)	6.35 (0.89)
Endorsement of past users	65.2	1 (2.35)	6.09 (0.94)
Informative pictures of accommodations	59.1	5 (3.03)	6.11 (0.95)
Trust seals of external validators	48.5	3 (2.44)	5.61 (0.99)
Possibility to communicate with staff members and users of the platform (e.g., Chat, Skype)	43.9	6 (3.07)	5.79 (0.87)
Different possibility to get in contact with platform operators (e.g., Blog, Social Media, Email)	42.4	8 (3.39)	5.71 (0.92)
Functioning of all links (no error messages, no page under construction)	37.9	7 (3.28)	5.86 (1.00)
Filter function in search tool for accommodations in working order	31.8	11 (3.90)	5.64 (1.02)
Appealing design and layout	30.3	10 (3.85)	5.44 (0.83)
Toll free number for help	22.7	9 (3.47)	5.50 (1.00)
Endorsement of newspapers	24.2	4 (2.94)	5.33 (1.00)
Presence of filter function in search tool for accommodations	9.1	12 (4.00)	5.50 (1.08)
Links to helpful external offers on site (e.g., car rental, restaurant suggestions)	6.1	13 (4.75)	5.36 (0.87)

*Note*: Only trust‐building measures that were chosen as one of the five most important measures by more than 5% of participants were taken into account, leaving 13 measures from the original 15 trust‐building measures for further analysis.

Based on the results of the pre‐test, we recreated a homepage of a P2P platform in the style of the platforms analysed in the pre‐test, including typical features such as a search mask. In the main study, participants were asked to imagine that a friend is thinking about booking accommodation for a vacation via the fictional platform HOLIDAY HOMES and they were tasked with examining the homepage of the platform. Afterwards, one of six versions of the homepage was randomly presented to the participants. All homepages were identical, differing only in the trust‐building measures presented and the information about the amount of regulation exercised by the platform. The manipulation of trust‐building measures and regulation exercised resulted in a 3 (information regarding security of payment versus reputation system versus no trust‐building measure) by 2 (low regulation vs. high regulation) design; see Appendix 3.

Participants either saw a chart showing the possibility of rating the accommodation after the stay (reputation), a chart indicating a secure payment system (information regarding security of payment) or a neutral chart with none of the above hints (no trust‐building measure); see Appendix 4. The manipulation of regulation was performed with text on the homepage. This text either stated that there would be checks of identity, reviews, contents and other consumer information on a sample basis (high regulation) or no checks would be undertaken (low regulation).

After participants saw the homepage, they could nominate the platform HOLIDAY HOMES for the fictional award “Trust and Safety”. They were told that this is an audience award for websites which have been evaluated by visitors to the homepage as remarkably trustworthy and secure. Next, the participants answered questions concerning their behavioural intention, comprising the intention of recommending the platform, registering on the website and booking accommodation via the platform (three items adapted from Bart et al., 2005, *α* = .90). Also, questions concerning trust (four items adapted from Bart et al., 2005; three items adapted from Pizzol et al., 2017, *α* = .97), risk perception (three items adapted from Corbitt et al., 2003, *α* = .74) and trustfulness (four items adapted from Cattell, 2001, *α* = .89) on a 7‐point Likert scale ranging from 1 (“I totally disagree”) to 7 (“I totally agree”) were included in the questionnaire. Furthermore, attitude towards and usage of online booking (three items adapted from Martínez‐López et al., 2005, *α* = .70) as well as experiences with P2P accommodation platforms were asked about (see full list of items in Appendix 6). After that, a manipulation check was implemented. Four different text passages with information about regulation were presented to the participants, who had to identify the text they had seen on the website. To check whether the manipulation of trust mechanisms worked out, three different charts were presented and participants had to indicate which one they had seen on the website. Finally, socio‐demographic data were assessed.

### 5.2 Results

First, a one‐way ANCOVA was performed to determine a difference between the six conditions regarding trust‐building measures and regulation on the intention to engage as the dependent variable controlling for participants' experience with P2P accommodation platforms. The results indicated no significant differences between groups (*F*(5,144) = 1.44, *p* = .214, *η*_p_^2^ = 0.05). The covariate experience had no significant influence on the dependent variable (*p* = .358). Furthermore, a one‐way ANCOVA was performed to test for significant differences between the groups on the dependent variable risk perception controlling for participants' experience. Again, the results did not show significant differences between the groups (*F*(5,144) = 1.75, *p* = .128, *η*_p_^2^ = 0.06). Participants' experience also had no significant influence (*p* = .677).

**FIGURE 1:**
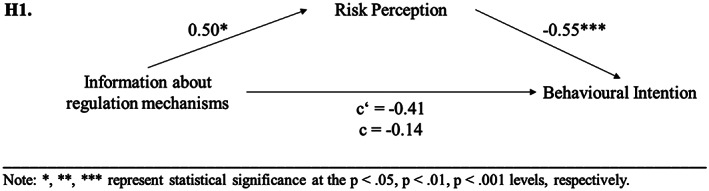
Experimental study 1: results of mediation analyses concerning H1.

In order to test hypothesis 1, a mediation analysis was performed with PROCESS (Hayes, 2017). In Step 1 of the mediation model, the regression of information about regulation mechanisms on behavioural intention, ignoring the mediator risk perception, was not significant (*b* = −0.41, *t*(148) = −1.69, *p* = .093). Step 2 showed that the regression of information about regulation mechanisms on the mediator (risk perception) was significant (*b* = 0.50, *t*(148) = 2.40, *p* < .05). Step 3 of the mediation process showed that the regression of risk perception (mediator) on behavioural intention was also significant (*b* = −0.55, *t*(147) = −6.33, *p* < .001). Step 4 of the analyses revealed that controlling for the mediator, information about regulation mechanisms was not a significant predictor of behavioural intention (*b* = −0.14, *t*(147) = −0.60, *p* = .553). The covariate experience did not significantly predict any of the variables in the mediation model. Thus, the results revealed that risk perception fully mediated the relationship between information about regulation mechanisms on behavioural intention, accepting H1 (see Figure 1).

**FIGURE 2:**
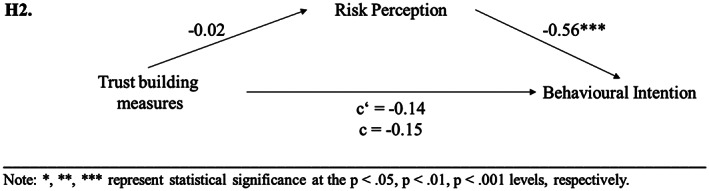
Experimental study 1: results of mediation analyses concerning H2.

A second mediation analysis was performed to test hypothesis 2. The regression of trust‐building measures on behavioural intention, ignoring the mediator risk perception, was insignificant (*b* = −0.14, *t*(148) = −0.93, *p* = .355). Step 2 showed that the regression of trust‐building measures on the mediator (risk perception) was also not significant (*b* = −0.02, *t*(148) = −0.12, *p* = .904). However, the mediation analysis revealed that the regression of risk perception (mediator) on behavioural intention was significant (*b* = −0.56, *t*(147) = −6.92, *p* < .001). Step 4 of the analysis showed that, when controlling for the mediator, trust‐building measures were not significant predictors of behavioural intention (*b* = −0.15, *t*(147) = −1.09, *p* = .278). Again, the covariate experience did not predict any of the variables in the mediation model. Thus, the results showed that the used trust‐building measures had neither a significant influence on risk perception as mediator, nor on behavioural intention; therefore, H2 was not supported (see Figure 2).

Besides the dependent variable of behavioural intention and thus, participants' willingness to engage in P2P sharing activities on the platform, participants were also asked whether they would care to nominate the platform HOLIDAY HOMES for the fictional award “Trust and Safety”. Thereby, we found that particularly participants in the condition of high regulation would nominate the platform for the award compared to participants in the condition of low regulation, independently of the trust‐building measures they found on the platforms' homepage (see Table 3 for detailed results).

**TABLE 3:** Percentages of (non‐) nominations for the platform HOLIDAY HOMES for the fictive award “Trust and Safety” per condition.

		Exercised regulation	Nomination	
			Yes (%)	No (%)
Trust‐building measures	Reputation system	Low regulation	26.7	73.3
		High regulation	48.3	51.7
	Information regarding security of payment	Low regulation	30.0	70.0
		High regulation	48.5	51.5
	No trust‐building measure	Low regulation	17.9	82.1
		High regulation	50.0	50.0

The results of the analyses supported hypothesis 1: Information about additional checks and monitoring on the website (high regulation) results in lower risk perception and higher behavioural intention of potential consumers (H1). In addition, higher regulation leads to more nominations for the fictional award “Trust and Safety”. However, in the current study we were not able to confirm hypothesis 2: no trust building measures, both information about security of payment as well as a reputation system, were found to significantly influence the levels of perceived risk or behavioural intention of potential consumers (H2).

## 6 EXPERIMENTAL STUDY 2

In order to verify the results of the experimental study 1, another study was conducted. Most importantly, this time the sample consists only of consumers, who had no prior experience with P2P accommodation platforms and thus, represent potential customers.

### 6.1 Method

#### 6.1.1 Sample

A representative sample from Austria (98.8%) was drawn for an online survey. Out of 300 participants who finished the questionnaire, for the subsequent analysis only participants who passed at least one of the manipulation checks, either on regulation or on trust‐building measures, were chosen. Thus, the final sample consists of 249 participants (47.8% men, *M*_age_ = 45.00 years, SD_age_ = 15.61, Range_age_ = 18–74). The majority of participants had either completed an apprenticeship (29.7%), had a secondary school completion certificate (23.7%) or a university degree (21.3%). None of the participants had experience with P2P accommodation platforms, neither as a host nor as a guest.

#### 6.1.2 Procedure

The procedure of the second experimental study followed the procedures of experimental study 1 with some slight changes. Again, the homepage of the fictional P2P platform HOLIDAY HOMES was used, but the website was simplified, text and information were reduced and the manipulation of trust‐building measures and regulation in a three (information regarding security of payment vs. reputation system vs. no trust‐building measure) by two (low regulation vs. high regulation) design was highlighted (see Appendix 3). One of the six versions of the homepage was randomly presented to the participants. Participants either saw a chart showing the possibility of rating the accommodation after the stay (reputation), a chart indicating a secure payment system (information regarding security of payment) or a neutral chart with none of the above hints (no trust‐building measure), see Appendix 5. The manipulation of regulation was performed with text on the homepage. This text either stated that there would be checks of identity, reviews, contents and other consumer information on a sample basis (high regulation) or no checks would be undertaken (low regulation).

After participants saw the homepage, they answered questions concerning their behavioural intention, comprising the intention to recommend the platform, register on the website and book accommodation via the platform (three items adapted from Bart et al., 2005, α = .92). Furthermore, questions concerning trust (four items adapted from Bart et al., 2005; three items adapted from Pizzol et al., 2017, *α* = .97), risk perception (three items adapted from Corbitt et al., 2003, *α* = .88), trustfulness (four items adapted from Cattell, 2001, *α* = .89) on a seven‐point Likert scale ranging from 1 (“I totally disagree”) to 7 (“I totally agree”) and the attitude towards and usage of online booking (3 items adapted from Martínez‐López et al., 2005, *α* = .80) (see full list of items in Appendix 6). After that, (i) a manipulation check for trust and (ii) a manipulation check for regulation was implemented. To check whether the manipulation of trust mechanisms worked out, three different charts were presented and participants had to indicate which one they had seen on the website. Two different text passages with information about regulation were presented to the participants, who had to identify the text they had seen on the website. Finally, socio‐demographic data were assessed.

### 6.2 Results

**FIGURE 3:**
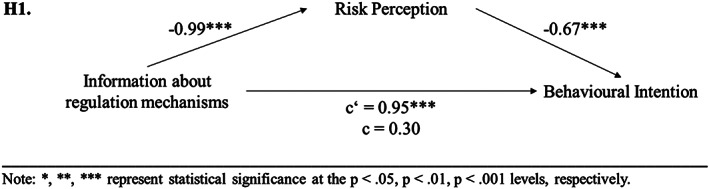
Experimental study 2: results of mediation analyses concerning H1.

**FIGURE 4:**
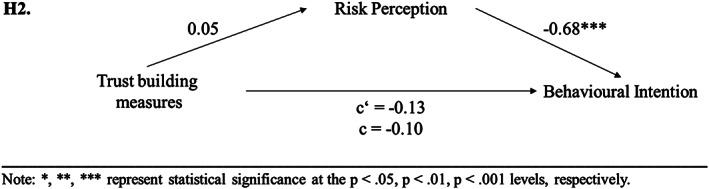
Experimental study 2: results of mediation analyses concerning H2.

First, we tested if the two groups (low regulation vs. high regulation) differed in their intention to engage. For the analyses, only the data of participants who passed the manipulation check on regulation were included (*N* = 206; 49.0% men, *M*_age_ = 45.43 years, SD_age_ = 15.60, Range_age_ = 18–74). An independent t‐test revealed that participants in the low regulation‐condition showed a significantly lower intention to engage (*M*_low reg_ = 3.36, SD_low reg_ = 1.74) than participants in the high regulation‐condition (*M*_high reg_ = 4.31, SD_high reg_ = 1.63; *t*(204)= −4.01, *p* < 0.001). The groups also differed significantly in their level of perceived risk (*t*(204) = 4.97, *p* < 0.001) with participants in the high regulation‐condition showing a lower level of perceived risk (*M*_high reg_ = 3.56, SD_high reg_ = 1.40; *M*_low reg_ = 4.54, SD_low reg_ = 1.40). Additionally, a mediation analysis was performed with PROCESS (Hayes, 2017). In Step 1 of the mediation model, the regression of information about regulation mechanisms on behavioural intention, ignoring the mediator risk perception, was significant (*b* = 0.95, *t*(204) = 3.94, *p* < .001). Step 2 showed that the regression of information about regulation mechanisms on the mediator (risk perception) was significant (*b* = −0.99, *t*(204) = −4.94, *p* < .001). Step 3 of the mediation process showed that the regression of risk perception (mediator) on behavioural intention was also significant (*b* = −0.67, *t*(203) = −8.84, *p* < .001). Step 4 of the analyses revealed that controlling for the mediator, information about regulation mechanisms was not a significant predictor of behavioural intention (*b* = 0.30, *t*(203) = 1.44, *p* = .153). Thus, the results revealed that risk perception fully mediated the relationship between information about regulation mechanisms on behavioural intention and, thereby, confirmed the result of experimental study 1 and allows for the acceptance of H1 (see Figure 3).An ANOVA was performed to test if the three groups (information regarding security of payment vs. reputation system versus no trust‐building measure) differed in their intention to engage. For the analyses, only the data of participants who passed the manipulation check on trust‐building measures were included (*N* = 169; 46.2% men, *M*_age_ = 43.89 years, SD_age_ = 15.86, Range_age_ = 18–74). The results showed no significant differences between groups (*F*(2,166) = 2.35, *p* = .099, *η*_p_^2^ = 0.03). The groups also did not differ significantly in their level of perceived risk (*F*(2,166) = 1.86, *p* = .159, *η*_p_^2^ = 0.02). A mediation analysis was performed to further test hypothesis 2. The regression of trust‐building measures on behavioural intention, ignoring the mediator risk perception, was not significant (*b* = −0.13, *t*(167) = −0.79, *p* = .432). Step 2 showed that the regression of trust‐building measures on the mediator (risk perception) was also not significant (*b* = 0.05, *t*(167) = 0.35, *p* = .730). The mediation analysis revealed that the regression of risk perception (mediator) on behavioural intention was significant (*b* = −0.68, *t*(166) = −7.47, *p* < .001). Step 4 of the analysis showed that controlling for the mediator, trust‐building measures were not significant predictors of behavioural intention (*b* = −0.10, *t*(166) = −0.71, *p* = .477). Thus, the results showed that the used trust‐building measures neither had a significant influence on risk perception as mediator, nor on behavioural intention; therefore, the results of experimental study 1 were confirmed and H2 was not supported (see Figure 4).

## 7 GENERAL DISCUSSION

Sharing private accommodation, cars, and other goods using online platforms is increasing in popularity, although P2P sharing via internet platforms bears risks for consumers. Recent unpleasant events relating to Airbnb accommodation sharing (cf. Hook, 2017; Wiliams, 2016) have unleashed a public debate on how to reduce risks for consumers. Whereas some argue that the organising platforms should rely on trust‐building measures implemented on their websites, particularly reputation systems, others call for stricter regulation. The current research tries to shed light on the question of how trust‐building measures and regulation of the sharing activity influences potential consumers' level of risk perception and their intention to use the service, focusing on online P2P accommodation platforms.

Results from a prestudy indicate that currently P2P accommodation platforms rely on the presentation of trust‐building measures and reputation systems on their websites. A content analysis of their terms and conditions reveals that regular monitoring or identity checks and checks of content are not carried out by the platforms. This is in line with earlier research on Airbnb (cf. Zervas et al., 2017) suggesting that the business models of P2P platforms currently operate with minimal regulatory controls. However, all platforms analysed in the current study reserve the right to exclude consumers. The experimental studies build on these results by manipulating the information displayed on different sharing platform homepages. Based on the results, we assume that regulations are the key factor in reducing risk perception levels and thereby increase potential consumers' willingness to engage in sharing activities managed by a P2P platform. On the contrary, trust‐building measures on a website were not found to play an important role in reducing potential consumers' level of perceived risk and therefore do not facilitate people's participation.

### 7.1 Theoretical implications

The current research adds to the literature on the relationship between risk, trust, and regulation. Previous research has often focused solely on one of these aspects. The implementation of trust‐building measures on websites has already been researched in the case of B2C online vendors (e.g., Casado‐Aranda et al., 2019; Kim & Peterson, 2017; Pengnate & Sarathy, 2017). However, to our knowledge the current paper is the first to apply these measures to the context of P2P sharing via an online platform. Our results show that trust‐building measures cannot be assumed to be effective in this context as well. This may be due to the point that the question of ownership is different for online vendors, such as companies that own the product, good or service of interest, whilst P2P platforms in the sharing economy only act as an intermediary between private persons. The question of how much risk is perceived by (potential) consumers of P2P accommodation websites and if trust and regulation can reduce it, is relevant to the platforms offering this service. Various scales have been developed for measuring risk perception of consumers (e.g., Ariffin et al., 2018; Weber et al., 2002). In our studies, we used the well‐established dimension performance risk of Corbitt et al. (2003). However, future research may want to use other means to test the risk perception of consumers in the context of the sharing economy in order to get a holistic picture of the phenomenon.

### 7.2 Practical implications

In practice, managerial strategies aiming to reach potential consumers of online offers within the sharing economy need to be customised for this specific online environment. Based on the impossibility of relying on physical and personal cues, such as a store or salesperson, the website of such platforms needs to provide a good impression and establish trust in the potential consumer. The current research has valuable implications for the marketing of P2P platforms: the way they act as an intermediary, enabling strangers worldwide to organise sharing, can either increase or reduce risks for consumers, e.g., by running background checks on new consumers. Currently, reputation systems are used as a universal remedy on P2P online sharing platforms (cf. Abrahao et al., 2017), which is supported by our research. Platforms thereby apply reputation systems as a form of trust‐building measure, which should increase trust in the service and reduce the level of perceived risk. Yet, assessments via reputation systems can be problematic; for instance, when untruthful feedback behaviour leads to positive review bias (Li et al., 2016). Doubts about the authenticity of reviews would negatively influence trust. Findings of Hartl et al. (2016) on collaborative consumption may also apply to digital platforms in the sharing economy: if consumers do not trust others, they call for regulation and governance. This may also be the case if digital platforms fail to establish trust. We advise marketing managers of P2P platforms to focus more on the prominent display of regulation mechanisms in order to mitigate potential consumers' perceived risk and increase their intention to use P2P platforms.

### 7.3 Limitations and future research directions

Besides their merits, the current studies also include some limitations. First, the majority of participants of experimental studies 1 and 2 were from Austria. The perception of risk and the importance of trust‐building and regulatory measures might vary for consumers of other cultural areas. As P2P sharing platforms are operating worldwide, cultural differences in the relevant variables should be addressed in future research on this topic in order to provide differentiated insights regarding the international consumer market P2P sharing platforms are operating in.

Second, the experimental studies help to identify relations between additional regulation in terms of monitoring and potential consumers' risk perception by using homepages of a fictitious P2P accommodation platform. However, sharing via online platforms involves a great variety of community features. The manipulation in the current research compromises information on regulation and trust building measures but cannot fully represent the complexity of sharing online in reality. For instance, in the experimental studies, participants were not able to interact with other consumers or employees of the platform or to experience an actual booking process. Nevertheless, the aim of the research is to investigate the risk perception of potential consumers whose first impression is mainly formed by the information provided on the homepage. If this very first impression cannot reduce potential consumers' risk perception, we assume that people will not dive deeper into the website and the platforms' offers. However, by implementing manipulation checks in order to test if participants noticed said information on the homepage, we revealed that especially when it comes to regulation, many participants in the condition of low regulation did not pass the manipulation check and indicated that they saw the information of high regulation on the website. Our pre‐test revealed that P2P accommodation platforms mainly rely on trust‐building measures and reputation systems on their websites instead of regular monitoring or identity checks and checks of content. Furthermore, they do not clearly state this on the homepage as we did in our experimental studies, but only in a terms and conditions section. This could potentially lead to a false impression of security for consumers, who presume that regulations are in place without looking for or rather noticing detailed information. However, future research on this topic should consider testing different approaches to visualise exercised regulation and build trust via the website in order to reveal new possibilities for P2P sharing platforms to reduce consumers' perceived risk and increase their intention to use the platform.

The current research focuses on potential consumers that may enter a P2P accommodation platform as a guest instead of a host. The chart used in the experimental study as a manipulation shows the procedure when booking accommodation and therefore is only applicable to potential guests. Risk perception as well as the reaction towards specific trust‐building and regulatory measures on a website may be different for people who plan to enter a P2P accommodation platform as host. For instance, potential hosts, unlike guests, are expected to present themselves and the accommodation offered in a favourable way to ensure booking (Tussyadiah & Park, 2018) and are confronted with the risk of physical damage to their property when hosting guests (Ranzini et al., 2020). They therefore may call for different kinds of support by the platform and need to be addressed with different trust‐building measures and regulatory mechanisms on a P2P platform. This difference should be addressed in future research. Further, ‘sharing economy’ has become a popular umbrella term for a variety of consumer activities. The current research focuses on P2P sharing of accommodation via platforms. Sharing a private flat or house involves certain risks that differ from sharing tools or clothes. To share one's own private home or to use the private accommodation of others may be perceived as an invasion of privacy. To be protected against any harm may therefore be of high relevance.

Furthermore, in both experimental studies, we focused on the influence of information about security of payment and no specific reputation system as trust‐building measures. Information about a secure payment system was found to be most important for participants in our pre‐test. However, future research should take a closer look at different trust‐building measures, like the endorsement of past users, informative pictures of accommodations and trust seals (Bart et al., 2005). In addition, different kinds of reputation systems on P2P platforms should be investigated as to their effectiveness in reducing the level of perceived risk and increasing the intention to recommend, join and use such a platform.

## FUNDING INFORMATION

This research was funded in whole, or in part, by the Austrian Science Fund (FWF) [P29693‐G29]. For the purpose of open access, the author has applied a CC BY public copyright licence to any Author Accepted Manuscript version arising from this submission.

## CONFLICT OF INTEREST

The authors declare that the research was conducted in the absence of any commercial or financial relationships that could be construed as a potential conflict of interest.

## Supporting information


**Appendix S1** Supporting Information.

## Data Availability

The data that support the findings of this study are available from the corresponding author upon reasonable request

## Footnotes

^1^

https://www.airbnb.at (Airbnb, 2022)

^2^

https://www.wimdu.at/ (Wimdu, 2018)

^3^

https://www.9flats.com/de (9flats, 2022)

^4^

https://gloveler.de/ (Gloveler, 2022)
