# Supplementary material for: Sharing on platforms: Reducing perceived risk for peer‐to‐peer platform consumers through trust‐building and regulation
Source: J Consum Behav. 2022 Jun 18;21(6):1255–67. doi: 10.1002/cb.2075 (PMC9821683; doi:10.1002/cb.2075)
Supplement: Supplementary file 1 — Appendix S1 Supporting Information. [file CB-21-1255-s001.docx]

**APPENDIX**

**APPENDIX 1.** Models of proposed variable relations

**
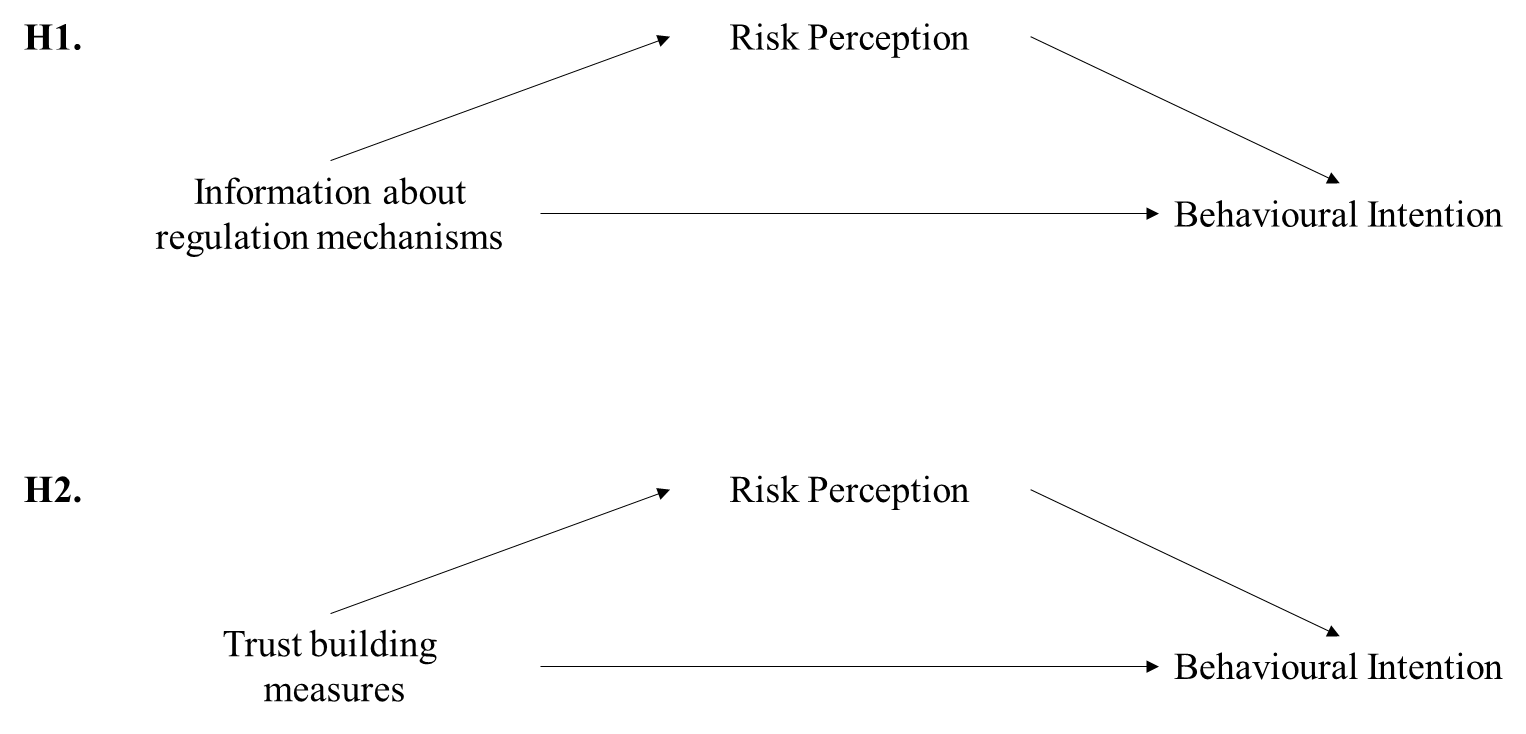
**

**APPENDIX 2.** Elaborated contents of general terms and conditions of P2P accommodation platforms

| 1. Control |
| --- |
| 1.1. Control |
| 1.2. No control of consumers |
| 1.2.1. No control of identity |
| 1.2.2. No control of reviews |
| 1.2.3. No control of consumer information |
| 1.3. No control of hosts |
| 2. Sanctions |
| 2.1. Sanctions against consumers |
| 2.1.1. Demand for modification of content |
| 2.1.2. Deletion of content |
| 2.1.3. Warning |
| 2.1.4. Complaint |
| 2.1.5. Monetary sanctions and reversals |
| 2.1.6. Limitation of usage, (temporary) closure of profile |
| 2.2. Sanctions against hosts |
| 2.3. Extent of sanction |
| 3. Rules |
| 3.1. Obligations of consumers |
| 3.1.1. Registration requirements |
| 3.1.2. Profile |
| 3.1.3. Veridical information |
| 3.1.4. Reviews |
| 3.1.5. Payment |
| 3.1.6. Illegal violation against laws |
| 3.1.7. Information obligation |
| 3.1.8. Seeking information |
| 3.1.9. Duty of disclosure |
| 3.2. General rules |
| 3.2.1. Mental or physical damage to others |
| 3.2.2. Additional guests |
| 3.2.3. Usage |
| 3.2.4. Spam |
| 3.2.5. Acceptance rate |
| 3.2.6. Rules of hosts |
| 3.2.7. Contents |
| 3.2.8. Violation of terms and policy |
| 3.2.9. Damage or disruption to platform |
| 3.2.10. Inappropriate usage |

**APPENDIX 3.** Overview of the 3 (trust-building measures) x 2 (regulation) design

|  |  | Exercised Regulation | |
| --- | --- | --- | --- |
|  |  | Low | High |
| Trust-building measures | Reputation system | Condition 1  (Reputation system,  low regulation) | Condition 2  (Reputation system,  high regulation) |
|  | Information regarding security of payment | Condition 3  (Security payment,  low regulation) | Condition 4  (Security payment,  high regulation) |
|  | No trust-building measure | Condition 5  (no trust-building,  low regulation) | Condition 6  (no trust-building,  high regulation) |

**APPENDIX 4.** Charts of the three different conditions concerning trust-building measures -Experimental Study 1


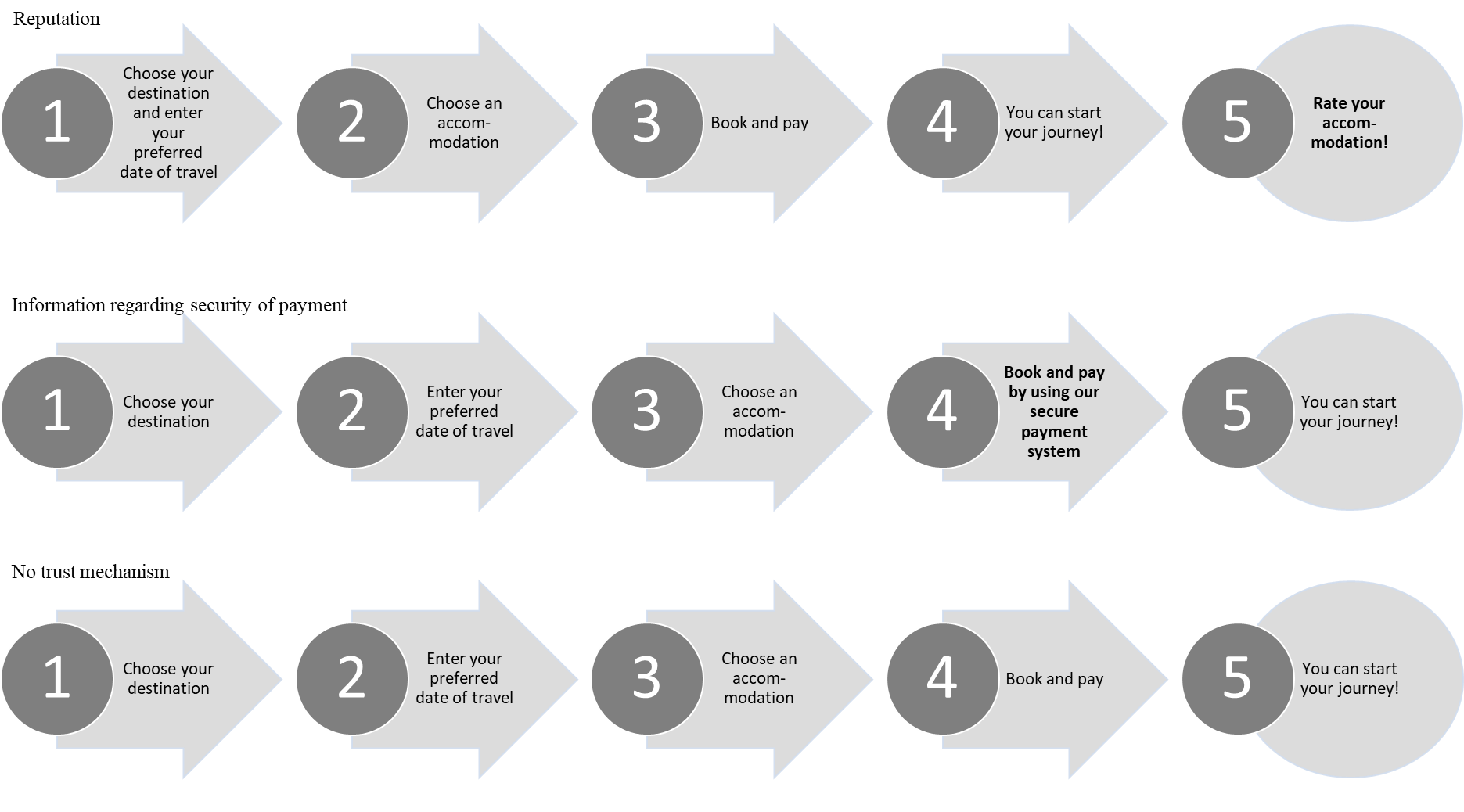


**APPENDIX 5.** Charts of the three different conditions concerning trust-building measures - Experimental Study 2

**
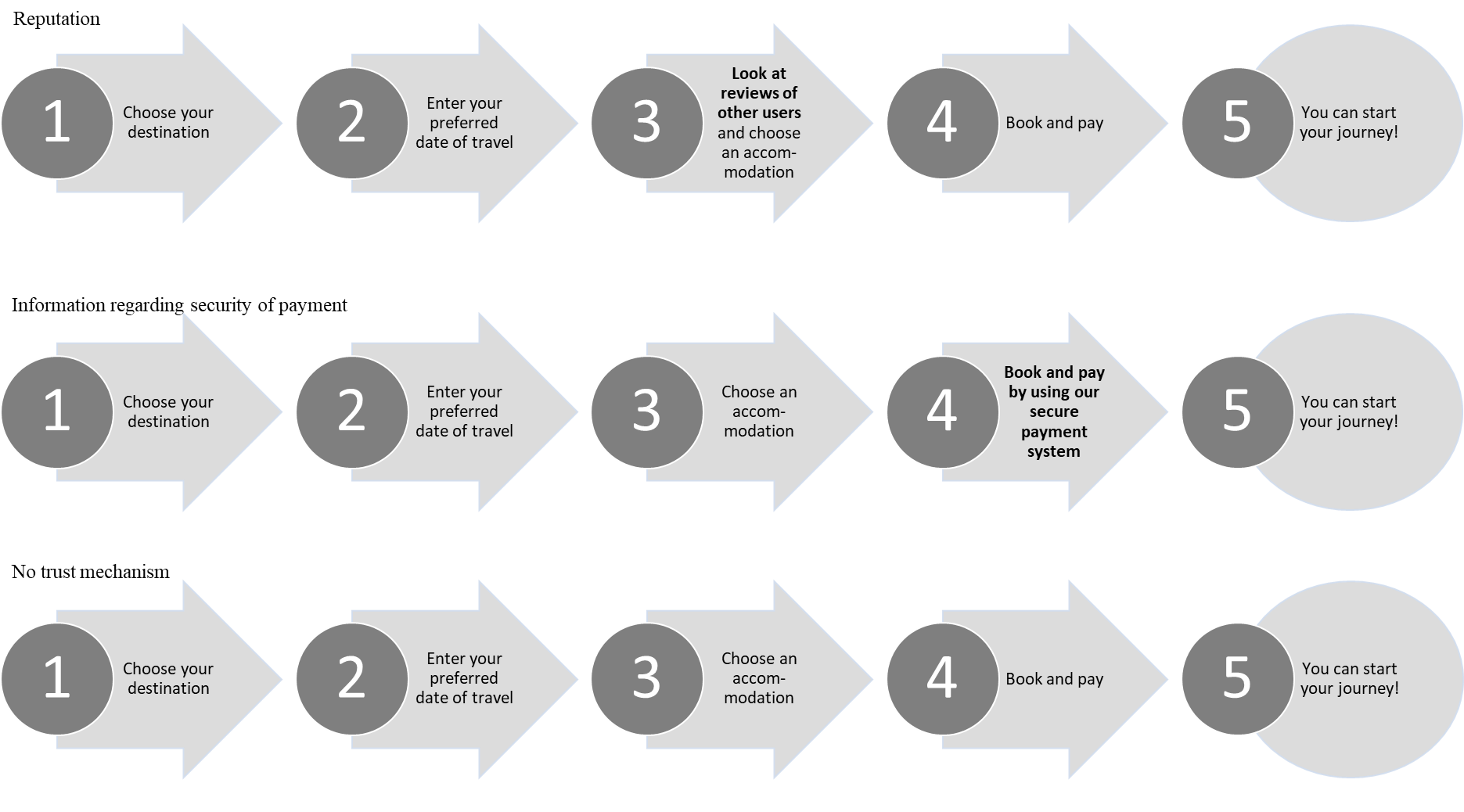
**

**APPENDIX 6.** Scales, Items and Cronbach’s α of questionnaires in Experimental Study 1 and 2.

| Scale | Source | Original items | Adapted items | Cronbach's α at Experimental Study 1 | Cronbach's α at Experimental Study 2 |
| --- | --- | --- | --- | --- | --- |
| **Behavioural Intention** | Bart et al., 2005 | I would purchase an item on this site. | I would book an accommodation on this website. | .90 | .92 |
|  |  | I would recommend this site to a friend. | I would recommend this website to a friend. |  |  |
|  |  | I would register at this site. | I would register at this website. |  |  |
|  |  | I am comfortable providing financial and personal information on this site.^1^ |  |  |  |
|  |  | I would book mark this site.^1^ |  |  |  |
| **Trust** | Bart et al., 2005 | This site appears to be more trustworthy than other sites I have visited. | This website of the company appears to be trustworthy. | .97 | .97 |
|  |  | The site represents a company or organization that will deliver on promises made. | The website represents a company that will deliver on promises made. |  |  |
|  |  | My overall trust in this site is | Overall my trust in this website is |  |  |
|  |  | My overall believability of the information on this site is | Overall the believability of the information on this website is |  |  |
|  |  | My overall confidence in the recommendations on this site is^2^ |  |  |  |
|  | Pizzol et al., 2017 | I trust the car sharing services I use. | I trust this platform. |  |  |
|  |  | The carsharing service is safe. | The offered service of this platform is safe. |  |  |
|  |  | I trust the car sharing operating model. | I trust in the service this platform is offering. |  |  |
| **Risk Perception** | Corbitt et al., 2003 |  | I believe that booking an accommodation via the platform HOLIDAY HOME is risky because the offered services… | .74 | .88 |
|  |  | I believe that on-line purchases are risky because the products/services delivered may fail to meet my expectations. | …may fail to meet my expectations. |  |  |
|  |  | I believe that on-line purchases are risky because the products/services delivered may be of inferior quality. | …may be of inferior quality. |  |  |
|  |  | I believe that on-line purchases are risky because the products/services delivered may be dangerous to use. | …may involve dangers. |  |  |
|  |  | I believe that on-line purchases are risky because the products/services may be available at a lower price somewhere else.^3^ |  |  |  |
|  |  | I believe that on-line purchases are risky because it may cause others to think less highly of me.^4^ |  |  |  |
|  |  | I believe that on-line purchases are risky because the products/services delivered may fail to fit well with my personal image or self-concept.^4^ |  |  |  |
|  |  | I believe that on-line purchases are risky in terms of time because the products/services delivered may fail to be delivered within the expected time frame.^3^ |  |  |  |
| **Trustfulness** | Cattell, 2001 | I trust in what people say. | I trust in what people say. | .89 | .89 |
|  |  | I trust in others. | I trust in others. |  |  |
|  |  | I believe that others have good intentions. | I believe that others have good intentions. |  |  |
|  |  | I believe that the majority of humans are moral. | I believe that in principle humans are moral. |  |  |
| **Attitude and usage  of online booking** | Martínez-López  et al., 2005 | Indicate your opinion towards the internet. | Please indicate your general opinion towards online booking. | .70 | .80 |
|  |  | negative ------- positive | negative ------- positive |  |  |
|  |  | I do not like it ------- I like it | I do not like it ------- I like it |  |  |
|  |  | Unfavourable ------- Favourable | I don't prefer it over other ways of booking (e.g., telephone, personally, etc.) ------- I prefer it over other ways of booking (e.g., telephone, personally, etc.) |  |  |

Note*:* Some of the original items were not used in the present studies for the following reasons:

^1^The item was not used in order to shorten the questionnaire.

^2^The item was not used, because instead of trust in other users’ recommendations, trust in the platform was the focus of the present studies.

^3^The item was not applicable to online P2P accommodation platforms.

^4^ The item was not used, because social and psychological risks were not the focus of the present studies.
